# Fatigue Analysis and Defect Size Evaluation of Filled NBR including Temperature Influence

**DOI:** 10.3390/ma15113745

**Published:** 2022-05-24

**Authors:** Jacopo Schieppati, Bernd Schrittesser, Stefano Tagliabue, Luca Andena, Armin Holzner, Jan Poduška, Gerald Pinter

**Affiliations:** 1 Polymer Competence Center Leoben GmbH, Roseggerstrasse 12, 8700 Leoben, Austria; b.schrittesser@scioflex.com; 2 SCIOFLEX GmbH, Opernring 1/R/748, 1010 Wien, Austria; 3 Dipartimento di Chimica, Materiali e Ingegneria Chimica “G. Natta”, Politecnico di Milano, Piazza Leonardo da Vinci 32, 20133 Milano, Italy; stefano.tagliabue@polimi.it (S.T.); luca.andena@polimi.it (L.A.); 4 Semperit Technische Produkte GmbH, Triester Bundesstrasse 26, 2632 Wimpassing, Austria; armin.holzner@semperitgroup.com; 5 Institute of Physics of Materials, Czech Academy of Sciences, 61662 Brno, Czech Republic; poduska@ipm.cz; 6 Department of Polymer Engineering and Science—Material Science and Testing of Polymers, Montanuniversität Leoben, Otto Glöckel-Strasse 2, 8700 Leoben, Austria; gerald.pinter@unileoben.ac.at

**Keywords:** fatigue, fracture mechanics, filled rubber, X-ray microtomography, defect size, temperature, J-integral

## Abstract

The fatigue behavior of a filled non-crystallizing elastomer was investigated on axisymmetric dumbbell specimens. By plotting relevant Wöhler curves, a power law behavior was found. In addition, temperature increases due to heat build-up were monitored. In order to distinguish between initiation and crack growth regimes, hysteresis curves, secant and dynamic moduli, dissipated and stored energies, and normalized minimum and maximum forces were analyzed. Even though indications related to material damaging were observed, a clear trend to recognize the initiation was not evident. Further details were revealed by considering a fracture mechanics. The analysis of the fracture surfaces evidenced the presence of three regions, associated to initiation, fatigue striation, and catastrophic failure. Additional fatigue tests were performed with samples in which a radial notch was introduced. This resulted in a reduction in lifetime by four orders of magnitude; nevertheless, the fracture surfaces revealed similar failure mechanisms. A fracture mechanics approach, which considered the effect of temperature, was adopted to calculate the critical defect size for fatigue, which was found to be approximately 9 μm. This value was then compared with the particle size distribution obtained through X-ray microcomputed tomography (μ-CT) of undamaged samples and it was found that the majority of the initial defects were indeed smaller than the calculated one. Finally, the evaluation of J-integral for both unnotched and notched dumbbells enabled the assessment of a geometry-independent correlation with fatigue life.

## 1. Introduction

Fatigue is one of the most common causes of the failure of elastomer components such as tires, dampers, seals, hydraulic hoses, and conveyor belts. In fact, rubber articles are commonly subjected to cyclic loads that may lead to the accumulation of damage and to the development and growth of cracks, which then lead to failure. Generally speaking, materials are assumed to naturally contain flaws and inhomogeneities that can lead, under specific loading conditions, to localized stress concentrations promoting the nucleation of cracks [1]. Indeed, fracture occurs due to defects, which may nucleate from inhomogeneities that grow when subjected to repeated loads and subsequently coalesce up to failure.

Continuum mechanics models can describe fatigue at a macroscopic scale, by considering the fatigue life exhibited at different stress or strain levels; however, obtaining the data necessary for the correct application of this approach is time-consuming, not to mention the large scatter in the data occurring due to the stochastic nature of the initiation.

An alternative approach is to consider stress intensification arising from the defects, using a fracture mechanics-based approach. The introduction of a dominant reproducible initial defect (i.e., notch) leads to the reduction of both scattering and testing time. Fatigue lifetime predictions can be obtained by summing up the initiation time and the integration over time (i.e., number of cycles) of the crack growth characteristic, by considering the initial defect size [1,2,3,4,5,6,7,8,9,10,11,12,13,14,15,16,17,18,19]. Inversely, knowing the fatigue lifetime, it is possible to estimate the size of the critical defect from which the failure started.

The typical dimension of the critical defects for different elastomers was found to be a few tens of μm [1,2,3,4,5,10,17]. Due to the complex formulations of typical rubber compounds, the identification of the origin of the critical defects that lead to failure is challenging. Commonly, the analysis of fatigue damage in rubbers is performed through techniques such as Scanning Electron Microscopy (SEM) [20,21,22,23,24,25,26,27,28,29,30] or X-ray microtomography (μ-CT) [18,30,31,32,33]. These methods represent powerful tools for the investigation of interrupted fatigue tests or for post-mortem analysis, giving insight to the defect population and its evolution during fatigue. A correct evaluation of defect size is fundamental for the accuracy of fatigue life prediction models.

In this study, the fatigue behavior of a filled acrylonitrile butadiene rubber (NBR) has been examined. This elastomer is particularly suitable for applications in the oil and gas industry due to its polarity, which makes it resistant to fuels. In such applications, the components are subject to alternating pressures that can cause fatigue damage and failure. Thus, the fatigue lifetimes of unnotched and notched axisymmetric dumbbell specimens were measured, and the behavior was further investigated by analyzing the evolution of surface temperatures, hysteresis curves, dynamic and secant moduli, and dissipated and stored energies. The fracture surfaces were also thoroughly analyzed.

Then, the assessment of the critical defect size for fatigue failure was performed using the fracture mechanics approach. This was done by considering the material fatigue crack growth [34] and taking into account the effect of temperature [35]. The calculated critical defect size was then compared with the dimensions of defects present in the undamaged specimen, which was evaluated through a μ-CT analysis. Finally, an equation for the evaluation of the J-integral was computed using a finite element method (FEM) simulation of the notched specimens, allowing to determine fatigue lifetime independently on the geometry.

## 2. Materials and Methods

### 2.1. Materials

The material used for this research is a commercial acrylonitrile butadiene rubber (NBR) filled with 42 parts per hundred rubber (phr) of carbon black (CB). Due to confidentiality agreements with the supplier, no further details about the formulation can be given.

### 2.2. Fatigue

**Figure 1:**
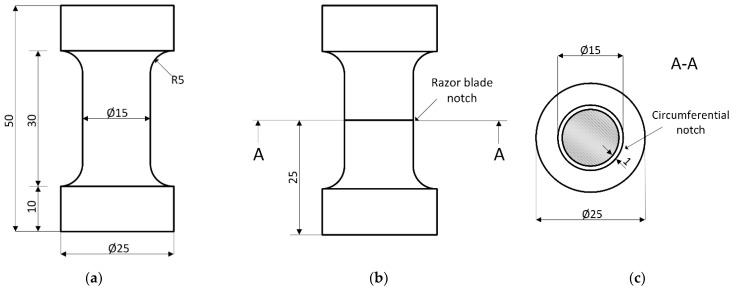
(**a**) Geometry and dimensions of the axisymmetric dumbbell specimens used in the fatigue experiments; (**b**) geometry of the Cracked Round Bar (CRB) specimen; (**c**) Section A-A of the CRB specimen with the circumferential notch.

Fatigue tests were conducted on axisymmetric dumbbell specimens (Figure 1a) on an MTS 858 Table Top System servohydraulic testing machine (MTS Systems GmbH, Eden Prairie, MN, USA). Hysteresis data were acquired every 100 cycles, and the force and displacement peaks were recorded on every cycle. A sinusoidal displacement waveform was used at a frequency of 4 Hz, with a load ratio *R_ε_* of 0.5. The maximum strains *ε_max_* were 5, 20, 50, 55, 60, and 65%. Complete failure of the specimen was considered as a lifetime criterion. Furthermore, the evolution of the surface temperature during the tests was monitored through a CT LT22 infrared sensor (Optris GmbH, Berlin, Germany) and recorded every 1 s.

Similarly, cyclic tests were performed on notched circular dumbbell specimens, denoted as Crack Round Bars—CRB (Figure 1b). According to ISO 18489 [36], an initial circumferential notch of approximately 1 mm depth (Figure 1c) was inserted at mid-height using a razor blade (thickness = 0.1 mm, tip-radius < 5 μm) mounted on a lathe rotating at 80 rpm. The specimens were cyclically loaded at maximum strains of 20, 30, and 50%, using the same frequency and load ratio used for the unnotched samples. Furthermore, the tests were monitored using a camera system CV-5701P by Keyence (Osaka, Japan) and pictures were recorded at every cycle.

### 2.3. Fracture Surface Analysis

An optical microscope Zeiss Stemi 2000C (Carl Zeiss AG, Oberkochen, Germany) was used for the fracture surface analysis.

### 2.4. X-ray Microtomography (μ-CT)

μ-CT scans of the dumbbell samples were acquired on a NSI X-25 inspection system (North Star Imaging, High Wycombe, UK). The X-ray source was operated at 40 kV and 100 µA, granting a magnification factor of 3.88 and a voxel resolution of 19 µm. It should be considered that the apparatus is capable of a nominal certified resolution of 2 µm [37]. The set of images was processed with a custom-made Matlab code (*Matlab R2020b*), performing binarization based on a combined global/local algorithm procedure [38] as a first step. The global algorithm adopted is the one developed by Otsu [39,40], which is able to distinguish between measurement artefacts and particles that are actually present inside the inspected specimen. Then, a local gradient-based watershed algorithm [41] was employed to better define the contour of the inspected particles. Following successful segmentation (i.e., separation of the voxels representing rubber and void, with Boolean values of 1 and 0, respectively), the code allowed particle detection by exploiting a spatial connectivity equal to 26, which means that two or more voxels belong to the same particle if they are in contact with at least one vertex. Each particle was then labelled, and its volume was computed as the sum of the connected full voxels (Boolean value equal to 1).

### 2.5. Evalaution of J-Integral Using Finite Element Method (FEM)

**Figure 2:**
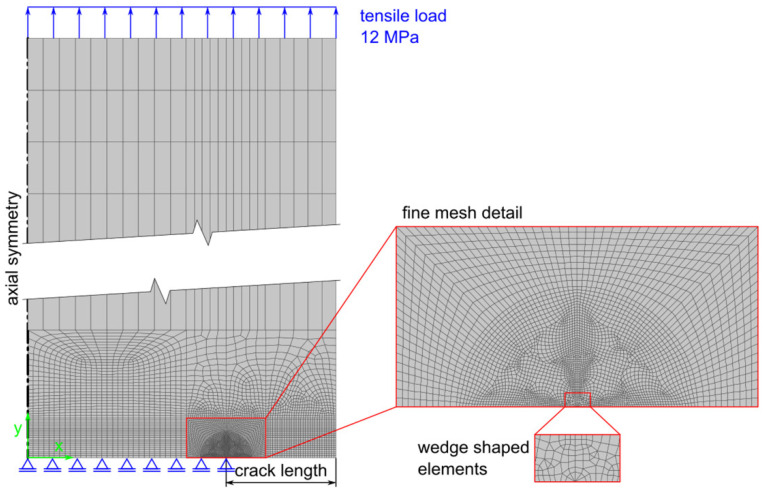
Mesh of the model used for the FEM calculation of the J-integral for CRB specimens.

FEM simulation of the CRB specimen behavior was carried out using a 2D axisymmetric model created in Ansys Parametric Design Language (APDL). The element type PLANE 183 was used, which is a higher order 2D, 8-node, or 6-node element [42]. Mapped mesh was used where possible. The mesh was made very fine in the vicinity of the crack tip with elements as small as 0.01 mm in the area closest to the crack tip. Moreover, special wedge-shaped crack tip elements were used at the crack tip. The rest of the model was meshed with a coarser mesh, with the largest elements up to 1.2 mm. The total number of elements was approximately 7000 for each of the modelled crack lengths. See Figure 2 where the mesh of the model for the crack length of 2.5 mm is depicted. A neo-Hookean incompressible hyperelastic solid was chosen for the material model; the shear modulus—obtained by fitting the uniaxial tensile test data—was 12.3 MPa, whereas the bulk modulus was not acquired as the material was assumed to be incompressible. Even though the rubbers are not strictly incompressible, this simplification does not make a big difference for the intended analysis. The goal of the simulation was to calculate values of the J-integral for various crack lengths and various loads. The calculation of the non-linear J-integral was performed through closed path integral calculation (CINT function in APDL).

## 3. Results

### 3.1. Fatigue of the Unnotched Dumbbell Specimens

**Figure 3:**
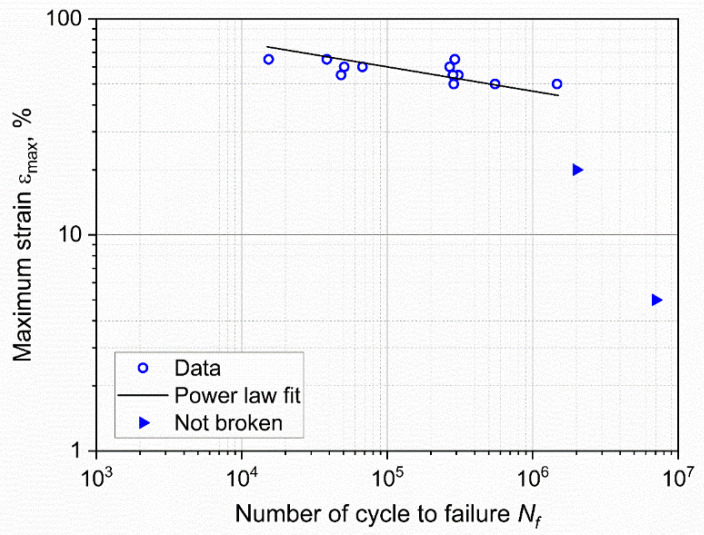
Wöhler curve obtained from axisymmetric dumbbell specimens of CB filled NBR at a frequency of 4 Hz and a load ratio *R_ε_* of 0.5. The fitting power law (Equation (1)) was obtained using ASTM E739–10.

The fatigue lifetime of the specimens subjected to cyclic loading is reported in Figure 3, in which the relevant *ε-N* or Wöhler curve [43] is represented. In particular, the maximum engineering strain *ε_max_* was selected as the representative loading parameter and plotted in double logarithmic scale against the number of cycles to failure, *N_f_*.

As depicted in the plot, a certain scattering of the results was found, which is typical for the fatigue of polymers and rubbers. The statistical analysis of the results was performed through the ASTM E739–10 [44], and the resulting fitting power law was found to be:

(1) $$\varepsilon_{max}=219\cdot N_{f}{}^{-0.11}.$$

Furthermore, two interrupted tests, for which no failure was recorded, are shown in Figure 3, and they correspond to *ε_max_* of 5% and 20%. In these two cases, the loads were so low that the failure would occur at a number of cycles that is well beyond the experimental window.

An alternative criterion for studying the fatigue nucleation of rubbers is to consider *N_i_*, corresponding to the number of cycles for the transition between the phase of initiation and crack growth. This is normally related to an abrupt variation of force (or stiffness) [45]. By applying this concept to the results previously shown, it was found that qualitatively, the plot was almost identical to the one reported in Figure 3 (provided in the Supplementary Material, Figure S1). In fact, the abrupt variation of force took place only in the last cycles (approximately 10 to 20 cycles). In actuality, this crack propagation phase corresponds to the macroscopic crack growth, which is visible to the naked eye. Generally, after the initial formation of cracks from inhomogeneities in the material, a small-scale growth of microcracks is present until macroscopic crack growth occurs, leading to a reduction of the ligament area and rapid failure.

**Figure 4:**
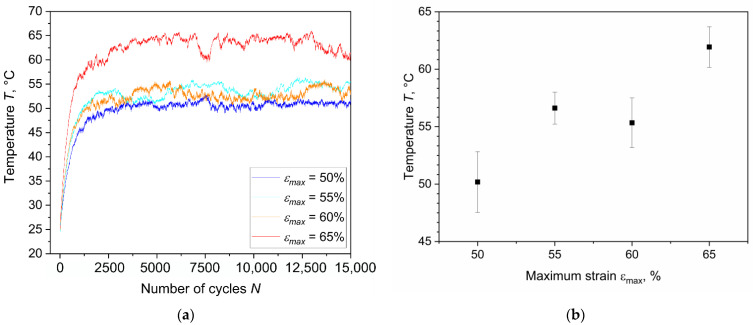
(**a**) Surface temperature evolution at different strains in the first 15,000 cycles of the fatigue experiments; (**b**) average values of plateau temperatures at different strains obtained during fatigue experiments from three samples per condition. The fatigue tests were implemented on axisymmetric dumbbell specimens of CB filled NBR at a frequency of 4 Hz and load ratio *R_ε_* of 0.5.

Further analysis of the fatigue tests led to the inspection of temperature evolution upon cyclic loading. In Figure 4a, the variation of the surface temperature for the first 15,000 cycles is shown. The temperature variation is a consequence of heat build-up; during every loading cycle, part of the mechanical energy is dissipated in the specimen volume and converted into heat. Due to the low thermal conductivity of rubbers [46,47], the internally generated heat slowly flows to the surrounding environment, and therefore, the temperature of the specimen significantly increases. This phenomenon strongly depends on the material compliance, on the frequency of oscillation, on the loading amplitude, and on the thickness of the component [48]. In fact, the rate of temperature variation $\dot{\Delta T}$ can be described as [49,50]:

(2) $$\dot{\Delta T}=\frac{\pi \cdot f\cdot \Delta \sigma^{2}\cdot D^{\prime \prime }\left(f,T\right)}{4\cdot c_{p}\cdot \rho },$$

where *f* is the frequency, Δ*σ* is the stress amplitude, *D*″ is the loss compliance, *c_p_* is the specific heat and *ρ* is the density; therefore, larger load amplitudes and frequencies will result in larger temperature increases. Indeed, the effect of increasing the loading amplitude is confirmed by the results reported in Figure 4a,b. The monitored temperature increases are consequences of the geometry of the specimen (i.e., the thickness) and the used frequency (4 Hz). In actuality, a decrease of these two variables could lead to the reduction of the temperature increase; however, thicker specimens are more representative for certain applications, and the axisymmetric dumbbell samples (or slight variations of it) represent a well-established geometry for fatigue characterization of rubbers [14,15,18,51,52,53,54,55,56,57,58,59,60,61,62]. The choice of the frequency has to match the requirement of being representative for the material application, and it is a tradeoff between testing time and temperature increases. Overall, with the used loading conditions, the surface temperatures increased in a range between 20 and 40 °C. Generally, the temperatures showed fluctuations related to the cooling system of the laboratory. Nevertheless, after approximately 2500–3000 cycles, the surface temperature became stable. As depicted in Figure 4b, higher maximum strains corresponded to higher temperatures in an almost linear relationship. The monitored temperature increases were so relevant that they must be considered during the analysis of fatigue results since such temperatures would affect the mechanical behavior of the material. In general, due to the entropic elasticity of the rubber matrix, an increase of stiffness would be expected; however, due to the presence of fillers and due to viscoelastic effects, the material would become softer at higher temperatures. Thus, the overall trend would be a trade-off between these opposite trends, and it would depend on the material composition. A reduction of stiffness at a higher temperature was previously reported for the material under investigation [35]. Nevertheless, the mechanical behavior is expected to be constant after reaching the plateau temperature.

**Figure 5:**
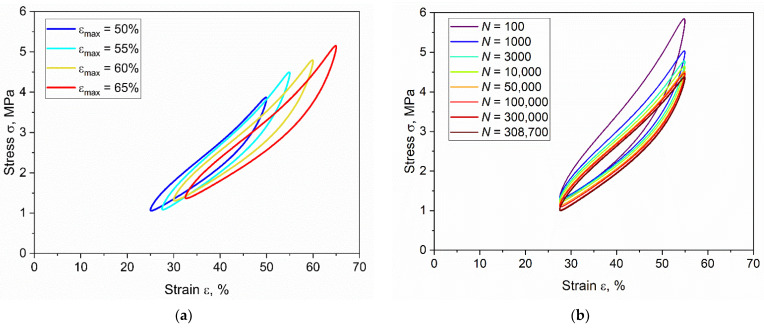
(**a**) Hysteresis curves at different maximum strains at 3000 cycles; (**b**) evolution of hysteresis curve at different number of cycles for maximum strain *ε_max_* of 55%. The fatigue tests were implemented on axisymmetric dumbbell specimens of CB filled NBR at frequency of 4 Hz and load ratio *R_ε_* of 0.5.

Further analyses of the fatigue data were implemented to investigate the distinct regimes of fatigue, namely, crack initiation and crack propagation. The hysteresis curves during fatigue tests for different strain levels are reported in Figure 5a, in terms of engineering stress and engineering strain at 3000 cycles. As depicted, higher strains corresponded to larger hysteresis. In Figure 5b, the evolution of the hysteresis curves during the tests is shown for a particular specimen (*ε_max_* = 55%) for a different number of cycles. A big variation of the hysteresis is present in the beginning of the test as a consequence of stress relaxation. The hysteresis seems to stabilize after approximately 3000 cycles, which corresponds to the stabilization of the surface temperatures. During the successive lifetime, the hysteresis variation was much smaller, which was a consequence of the combination of both stress relaxation and material damage accumulation.

**Figure 6:**
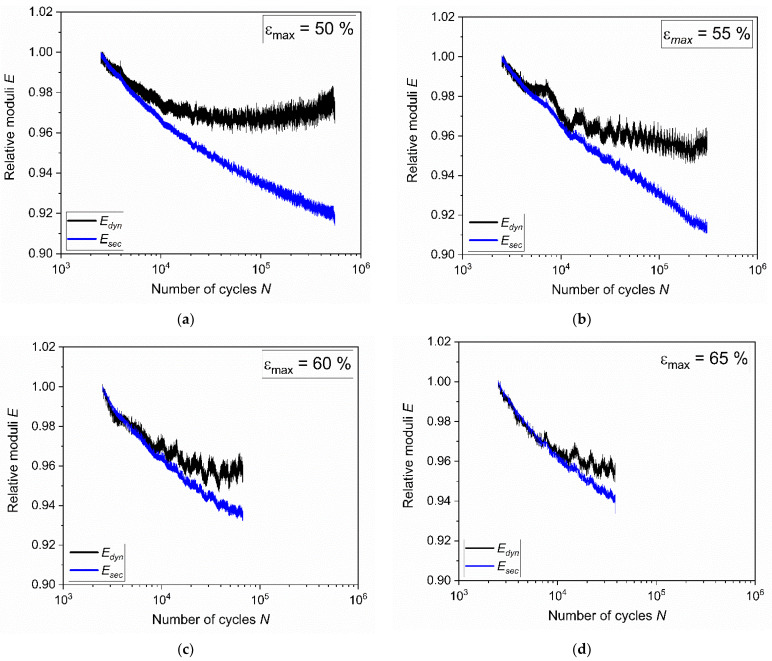
Dynamic (*E_dyn_*) and secant (*E_sec_*) moduli evaluated from hysteresis at maximum strain of (**a**) 50%, (**b**) 55%, (**c**) 60%, and (**d**) 65%, respectively. The fatigue tests were implemented on axisymmetric dumbbell specimens of CB filled NBR at a frequency of 4 Hz and a load ratio *R_ε_* of 0.5.

For a better understanding of the results, the hysteresis curves were analyzed considering two parameters, the secant *E_sec_* and the dynamic *E_dyn_* modulus that can be calculated according to Equations (3) and (4) [63,64]:

(3) $$E_{sec}=\frac{\sigma_{max}}{\varepsilon_{max}},$$

(4) $$E_{dyn}=\left|\frac{\sigma_{max}-\sigma_{min}}{\varepsilon_{max}-\varepsilon_{max}}\right|,$$

where *σ_max_* and *σ_min_* represent the maximum and minimum stresses and *ε_max_* and *ε_min_* are the maximum and minimum strain, respectively. Although the dynamic modulus is correlated with the instantaneous material response, the variation of the secant modulus is related to viscoelastic effects and to the accumulation of damage in the material [64,65]. Equations (3) and (4) are probably not able to fully describe the highly non-linear mechanical behavior of elastomers; however, for a first approximation, their trends for the number of cycles may provide interesting insights about fatigue. Due to the large variation in temperature at the beginning of the tests, both moduli initially showed large drops. For a better comparison, the values were normalized with respect to those at 2500 cycles (i.e., once temperature and mechanical behavior became stable). The results at the different strains are reported in Figure 6. In all cases, it is possible to observe a similar initial drop for both moduli. Subsequently, *E_dyn_* showed a lower rate of reduction and became more stable—even slight increases were monitored; therefore, the material stiffness remained essentially constant in the last stages of fatigue. The initial decrease and the successive stabilization were associated with cyclic stress softening [66], which seems to be connected to the breakdown and reformation of filler–molecule interactions [67]. On the other hand, *E_sec_* shows a steady drop for the entire test duration, as a consequence of the continuous stress relaxation and accumulation of damage during the test.

**Figure 7:**
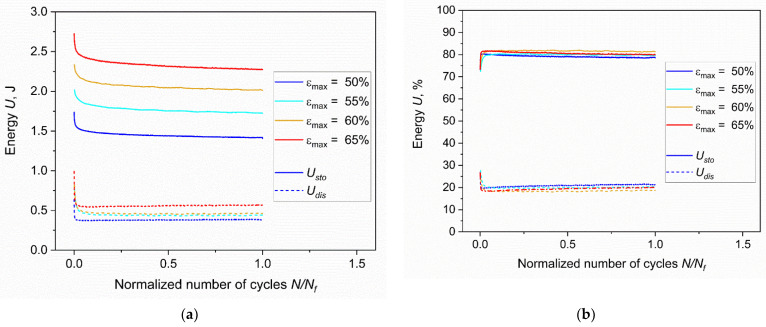
(**a**) Stored (*U_sto_*) and dissipated (*U_dis_*) energies at different strains against the normalized number of cycles; (**b**) percentage stored and dissipated energies at different strains against the normalized number of cycles. The fatigue tests were implemented on axisymmetric dumbbell specimens of CB filled NBR at a frequency of 4 Hz and a load ratio *R_ε_* of 0.5.

Subsequently, the energies were analyzed by integrating the area of the force-displacement curves from the hysteresis curves previously discussed. In this sense, it was possible to distinguish between the dissipated energy, *U_dis_*, as the area inside the hysteresis loop, and the stored energy, *U_sto_*, as the area below the unloading curve. For a proper comparison, the evolution of the energies was plotted as a function of the normalized number of cycles (currently elapsed number of cycles, *N*, divided by the number of cycles to failure, *N_f_*) as reported in Figure 7a. These results originate from the behavior already described for the hysteresis curves: in all the testing configurations, a drop in both energies was observed during the first cycles. As previously discussed, this effect is mainly a consequence of stress relaxation. After the drop, the values of *U_dis_* are then substantially stabilized for plateau values. The stored energy *U_sto_* instead kept decreasing with the number of cycles, albeit slightly, due to energy dissipation caused by material damage. On the other hand, the dissipated energies moderately increased, evidencing the increase in damage in the material. Moreover, a clear trend with respect to the maximum strains is present: both dissipated and stored energies were higher for higher strains; however, this description is different when considering the percentage of energy, which is the normalization with respect to the total energy, *U_tot_*, calculated as the sum of *U_dis_* and *U_sto_*. In fact, as reported in Figure 7b, the percentage energies were very similar for different strain levels and for the whole duration of the tests, maintaining values of approximately 20% for the dissipated energy and 80% for the stored one.

**Figure 8:**
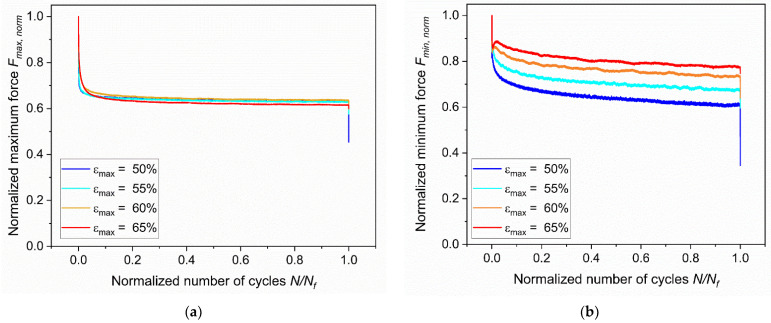
(**a**) Normalized maximum force at different strains against normalized number of cycles. (**b**) Normalized minimum force at different strains against the normalized number of cycles. The fatigue tests were implemented on axisymmetric dumbbell specimens of CB filled NBR at a frequency of 4 Hz and a load ratio *R_ε_* of 0.5.

Fatigue data were further analyzed by considering the peak values of the forces detected during the measurements. For a clearer comparison at different loading conditions, the maximum force, *F_max_*, and the minimum force, *F_min_,* were normalized with respect to their respective initial values and they were plotted against the normalized number of cycles (Figure 8a,b). Due to stress relaxation, the maximum and minimum forces showed a considerable variation in the first cycles, until consistent stress relaxation occurs in the specimen. After the initial drop, the values were constantly decreasing as a consequence of both stress relaxation and damage accumulation. In this region, the reduction extent depended on the loading conditions, but it was generally limited. Ultimately, a final major drop was present, which was related to the macroscopic crack growth and consequence of the reduction of the specimens’ ligament section. As depicted in Figure 8a, the maximum forces decreased at the very beginning of the tests by about one third and stabilized up to the final failure of the specimen, regardless of the strain level. Conversely, a different behavior was found for the minimum force. It is evident from the plot in Figure 8b that after the initial drop, the minimum forces are different for distinct strain levels: larger drops were monitored for lower values of maximum strain. Furthermore, small peaks were observed after the initial drop, which are more evident for larger maximum strains in particular.

Therefore, several parameters showed a reduction during the cycles, which was related to relaxation and material damage. These changes could be observed from the early stages of fatigue, suggesting a slow crack growth process; however, clear evidence to identify a transition between the regimes of crack initiation and crack growth were not found. Thus, further investigations were performed considering a fracture mechanics-based approach.

### 3.2. Fracture Surface Analysis of the Unnotched Dumbbell Specimens

**Figure 9:**
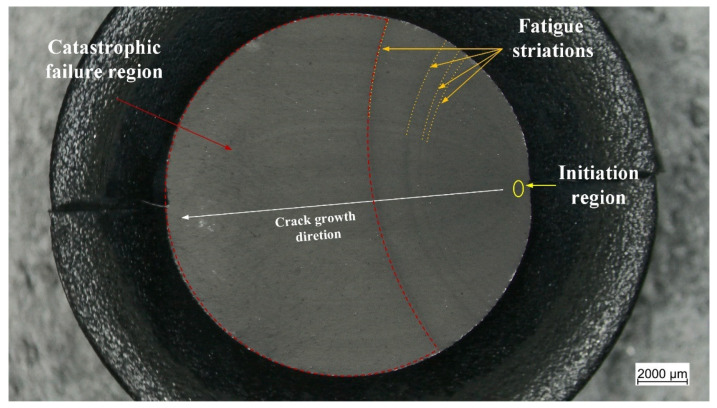
Light microscope picture of the fracture surface of an axisymmetric dumbbell specimen of CB filled NBR after fatigue testing. Three regions are displayed: initiation region (yellow), fatigue striation (orange), and catastrophic failure (red). The test was performed at a frequency of 4 Hz and a load ratio *R_ε_* of 0.5.

**Figure 10:**
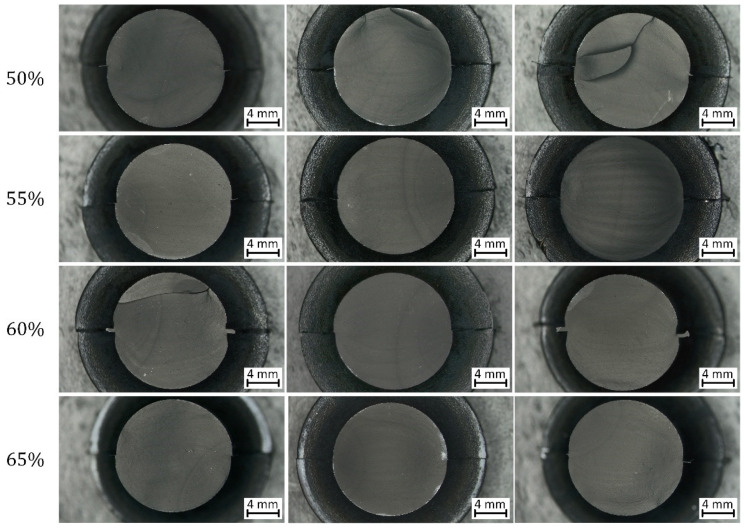
Light microscope pictures of fracture surfaces of axisymmetric dumbbell specimens of CB with NBR at different strains (the correspondent maximum strain is reported on the left). The tests were performed at a frequency of 4 Hz and a load ratio *R_ε_* of 0.5.

**Figure 11:**
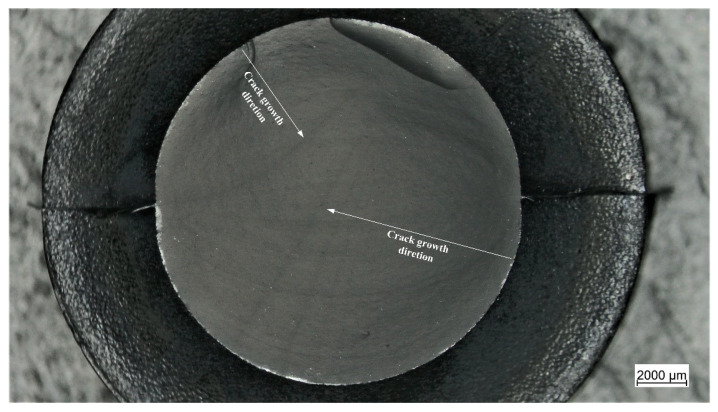
Light microscope picture of the fracture surface of an axisymmetric dumbbell specimen of CB filled NBR after fatigue testing in which two fronts of macroscopic fatigue crack growth are present (displayed by the arrows). The test was performed at a frequency of 4 Hz and a load ratio *Rε* of 0.5.

Subsequently, the fracture surfaces of the specimens were observed under the light microscope. The typical appearance of the fracture surfaces is shown and described in Figure 9. The most evident characteristic is the presence of fatigue striations which display an increasing distance from each other as they move farther from the initiation area. Normally, fatigue striations were observed for up to half of the surface area, whereas the remaining section corresponded to the catastrophic failure. An overview of the fracture surfaces at different strains is reported in Figure 10. Generally, failure occurred perpendicularly to the loading direction, with quite flat fracture surfaces, and the features displayed in Figure 9 were the most frequently observed with evident fatigue striation in all cases; however, in some cases, the fracture surfaces results were more complex, with evident macroscopic cracks out of the plane of the fracture surface at *ε_max_* of 50% and 60%. Such kinds of crack deviation could be related to local inhomogeneities in the material such as large agglomerates of carbon black. Moreover, at the lowest strain (*ε_max_* of 50%), fatigue striations in two opposite directions were observed (Figure 11), suggesting that the initiation is taking place independently in different areas and that at lower strains, macroscopic crack growth took place in different directions. The single crack direction at larger strains suggests that due to the larger energy and stress intensification involved, once the crack began to form, it became the predominant crack.

### 3.3. Fatigue of the Cracked Round Bar Specimens

Furthermore, fatigue measurements on the Crack Round Bar (CRB) were conducted. The CRB geometry (a dumbbell with a circumferential notch in the middle) was developed for accelerated testing of quasi-brittle failure resistance of polymers, elastomers, and thermoplastic elastomers [68,69,70]. In principle, due to the presence of the notch, this specimen geometry would allow the crack length and crack growth rate to be measured using a Paris approach, focusing on the crack propagation regime; however, due to the cylindrical geometry, the crack front results are complex, and it is difficult to assess crack length measurements through optical methods. More accurate results could be obtained by using extensometers and retrieving the crack length through a calibration between Crack Opening Displacement (COD) and the actual crack length [71]; however, due to the large strain involved, this approach has limitations for elastomers.

**Figure 12:**
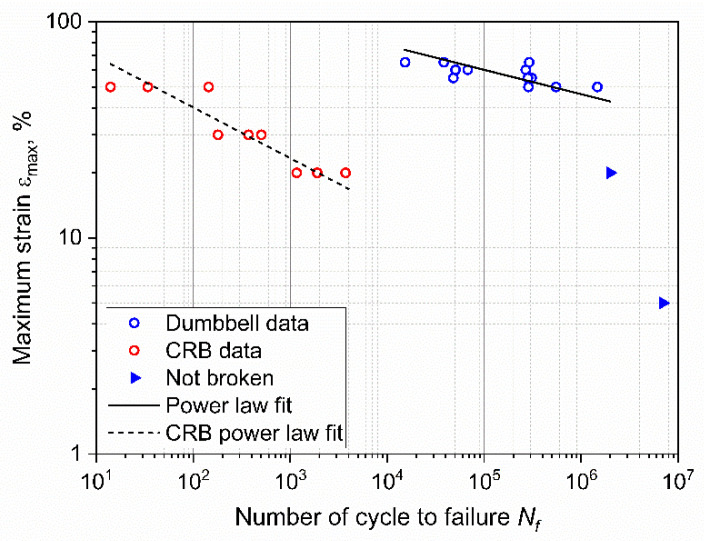
Wöhler curve obtained from axisymmetric dumbbell specimens (blue) and CRB (red) of CB filled NBR at a frequency of 4 Hz and a load ratio *R_ε_* of 0.5. The fitting power laws of the axisymmetric dumbbell specimen (continuous line) and of CRB (dashed line) were obtained using ASTM E739–10.

The results of fatigue tests obtained on CRB specimens in comparison with the results of standard specimens are shown in Figure 12. As depicted, a general reduction in lifetime was found for CRB as a consequence of the introduction of the notch. A reduction of about four orders of magnitude of the fatigue lifetime at *ε_max_* of 50% was recorded. Furthermore, in contrast to the results of unnotched axisymmetric dumbbells, failure was observed even at 20% of maximum (nominal) strain. The fitting power law from the statistical analysis of the results, performed through the ASTM E739–10 was found to be:

(5) $$\varepsilon_{max}=119\cdot N_{f}{}^{-0.24}.$$

**Figure 13:**
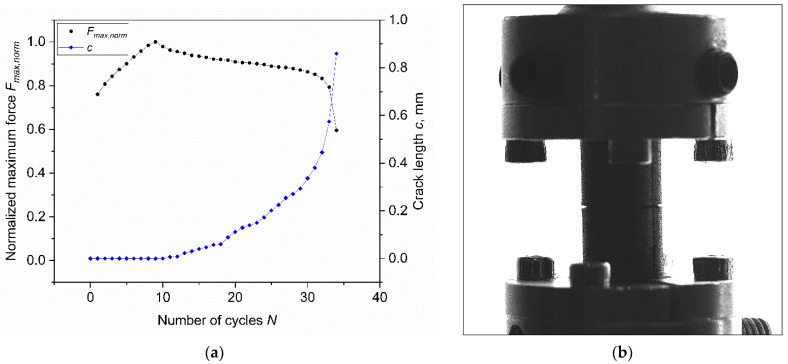
(**a**) Normalized maximum force (circles) and crack length (diamonds) against number of cycles for CRB specimens obtained from axisymmetric dumbbell specimens of CB filled NBR. (**b**) Picture of the tested CRB at *N* = 30; on both sides, the crack opening can be recognized. The test was performed at the maximum strain of 50% at a frequency of 4 Hz and a load ratio *R_ε_* of 0.5.

Moreover, the variations of both the normalized maximum force and crack length during the test with CRB geometry were analyzed and reported in Figure 13a. It is worth noting that the machine reached such high strains with a delay; since a stable deformation was observed after a transient period of eight cycles, the maximum force was attained after nine cycles. Considering the cylindrical symmetry of the samples, the crack length was approximated as the projection of the actual radial crack. In addition, the reported crack length was considered as the sum of the crack lengths observed on both sides of the specimen in the camera record (see the picture in Figure 13b). To have a proper measurement of the crack evolution, more cameras would be needed; however, this approximation is considered sufficiently accurate to monitor the evolution of the specimen’s ligament section. As depicted in Figure 13a, the larger crack increase was observed at about 30 cycles, a time at which an abrupt variation of the maximum force was also recorded; nevertheless, a smaller crack length increase was present even before this stage. In actuality, the crack starts to grow immediately after the application of the load—at the ninth cycle after stable deformation is reached. On the other hand, in unnotched specimens, the microscopic crack growth occurred after initiation.

### 3.4. Fracture Surface Analysis of the Cracked Round Bar Specimens

**Figure 14:**
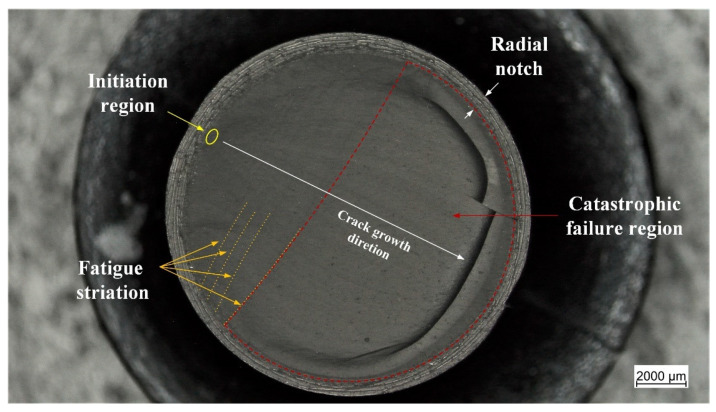
Light microscope picture of the fracture surface of a notched axisymmetric dumbbell specimen (CRB) of CB filled NBR after fatigue testing. At the specimen edge the radial notch can be observed, and a further three regions are displayed: initiation region (yellow), fatigue striation (orange)—corresponding to macroscopic fatigue crack growth—and catastrophic failure (red). The test was performed at a frequency of 4 Hz and a load ratio *R_ε_* 0.5.

Additionally, the fracture surfaces of the CRB specimen were analyzed and a representative example is reported in Figure 14. There are several similarities in the failure mechanism when compared with the unnotched kind in Figure 9. In addition to the region of radial notching, the fracture surfaces of CRBs also displayed the three aforementioned regions of initiation, fatigue striation, and catastrophic failure. Moreover, even in this case, the fatigue striations show increasing distances moving from the initiation region, and in correlation with the increasing crack length reported in Figure 13a. Overall, these results suggest that a similar damage mechanism is maintained, and that the introduction of the artificial notch is equivalent to a specimen with a larger initial defect in an unnotched specimen.

### 3.5. Defect Size Assesment

As already mentioned, the fatigue lifetime can be correlated with the crack growth by considering the initial size of defects that cause fatigue failure. In fact, by considering the defect size *c*_0_, it is possible to connect the failure of the material to defects and inhomogeneities naturally present in the material.

In order to obtain the value *c*_0_, the integration of the crack growth characteristic is required. Generally, the fatigue crack growth of rubbers within the stable regime can be described using the Paris law [5]:

(6) $$\frac{dc}{dN}=CG^{m},$$

where *dc/dN* represents the crack growth rate, *G* represents the tearing energy, and *C* and *m* are materials constants. For small cracks, the energy release rate (i.e., tearing energy) can be factored into strain energy density and crack size [2] and its estimation can be given by:

(7) G=2·k(λ)·W·c,

where *W* is the strain energy density and *c* is the crack length; *k*(*λ*) is a function of the stretch ratio [72]. In actuality, Equation (7) represents the tearing energy for single edge notch specimens [3]; nevertheless, it is a good approximation for small cracks in terms of initial defects. Moreover, the strain energy density *W* was evaluated considering the area under the loading curve by numerical integration of hysteresis data as:

(8) $$W=\int \sigma \cdot d\varepsilon =\int \frac{F}{A}\cdot \frac{ds}{s}=\frac{1}{V}\int_{s_{min}}^{s_{max}}F\cdot ds.$$

where *σ* is the engineering stress, *ε* is the engineering strain, *F* is the force, *A* the resistant area of the specimen, *s* is the displacement, *s_min_* and *s_max_* are the minimum and the maximum displacements, respectively, and *V* is the volume of the specimen. By combining Equations (6) and (7), and integrating the number of cycles for failure, *N_f_*, can be obtained:

(9) $$N_{f}=\frac{1}{\left(m-1\right)\cdot C{\left(2kW\right)}^{m}}\cdot \left(\frac{1}{c_{0}^{m-1}}-\frac{1}{c_{f}^{m-1}}\right).$$

Assuming that the initial defects size *c*_0_ is much smaller than the final crack length *c_f_*, this last contribution can be neglected, obtaining:

(10) $$N_{f}=\frac{1}{\left(m-1\right)\cdot C{\left(2kW\right)}^{m}}\cdot \frac{1}{c_{0}^{m-1}}.$$

This equation can be used to calculate the number of cycles to failure as a consequence of the growth of a pre-existing defect with the dimension *c*_0_. Alternatively, Equation (10) can be reversed to evaluate the effective critical defect size from which the failure originated. It is worth noting that for the evaluation of the overall lifetime, this approach for elastomers often neglects the initiation time; however, this is justified considering what was found through the analysis of the evolution of defects during fatigue, which investigated for filled polychloroprene rubber (CR) through μ-CT [31,32], and for filled natural rubber (NR) through SEM analysis [28,29]. It was shown that cracks are initiated in the early stages of fatigue tests and that the number of defects mainly increase within the first 10% of fatigue life. In the successive stages, the initiated defects slowly increased in size, whereas the number of cracks is more stable: it constantly increases but with a lower rate of nucleation. Therefore, the majority of the lifetime of rubbers is governed by crack growth and the initiation time can be neglected.

**Figure 15:**
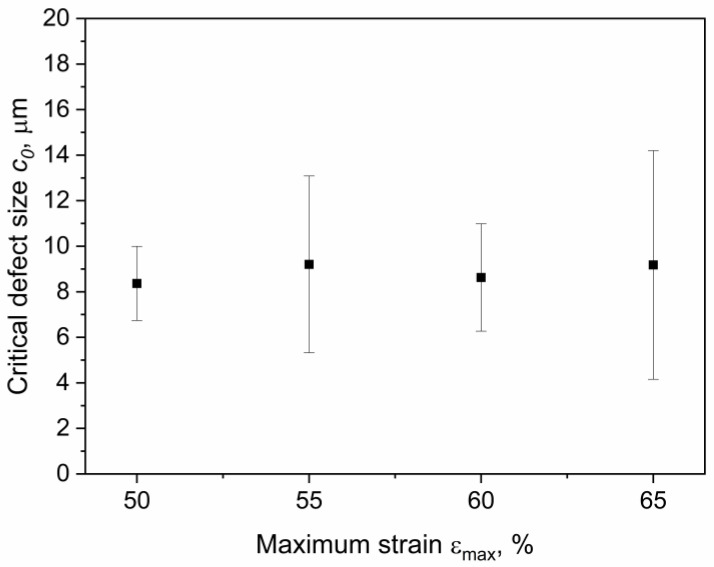
Critical defect size *c*_0_ at different strains evaluated from fatigue data using Equation (10). The fatigue tests were implemented on axisymmetric dumbbell specimens of CB filled NBR at a frequency of 4 Hz and a load ratio *R_ε_* of 0.5.

The effective critical defect size *c*_0_ was calculated by reversing Equation (10) and using the number of cycles to failure *N_f_*, as reported in Figure 3. The values of *k* and *W* were calculated for the corresponding strain levels. In particular, the strain energy density for each specimen was evaluated through Equation (8) at the beginning of the test. As for the coefficients *m* and *C* of the Paris law, these were evaluated from the data and the fatigue master curve shifting procedure was previously reported in [35]. In that work, fatigue crack growth characteristics at different temperatures (and at the same frequency and load ratio as in the work presented here) were analyzed, and a fatigue master curve based on the temperature dependence of the loss modulus was constructed. Using this procedure, horizontal shift factors were evaluated for the temperatures measured during the testing of axisymmetric dumbbells, allowing for the calculation of *m* and *C* for each test, while accounting for the influence of temperature on these parameters. An overview of all the values used for the calculation of *c*_0_ are reported in Table 1. Using these parameters, the effective critical defect sizes were calculated, and the values at different strains are reported in Figure 15. As depicted, the values *c*_0_ were found to be in the range between 4 and 14 μm for the different maximum strains. The values of the critical defect size are similar, regardless of the applied strain level: this points towards the conclusion that the defect size is an intrinsic value related to the material. The reason for the different degrees of dispersion in the results at different strains was mainly related to the large scattering of the fatigue data at the same strain values (see Table 1). Due to the independence of the size on the strain, it is possible to consider the mean value for all specimens: the effective critical defect size *c*_0_ was 9 ± 3 μm.

**Figure 16:**
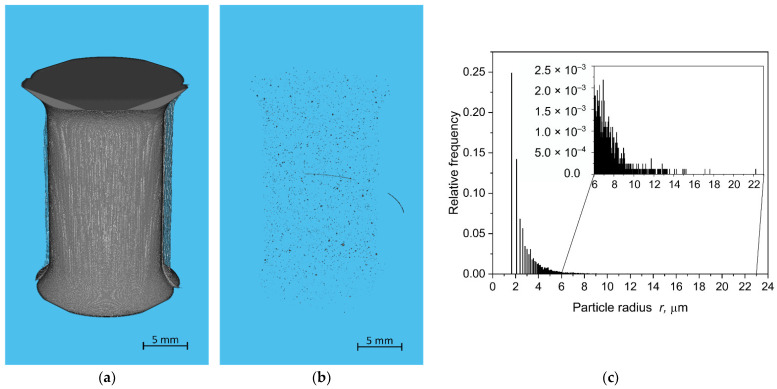
(**a**) Volume reconstruction of an undamaged (i.e., not yet loaded and cycled) axisymmetric dumbbell specimen obtained from μ-CT. (**b**) Cavity distribution in the reconstructed volume obtained from μ-CT with a connectivity equal to 26. (**c**) Particle size distribution considered as the spherical radius of the cavities.

Moreover, the initial defect size of the axisymmetric dumbbell specimens was evaluated through the reconstruction of μ-CT (Figure 16a). From the reconstructed volume, naturally present defects have been identified (Figure 16b) and their volumes have been determined. In order to have a direct comparison with the critical defect size *c*_0_, the cavities have been approximated as spheres and their radii have been calculated. The particle size distribution is displayed in Figure 16c, in which the relative frequency for each value of the particle radius is reported. As depicted, the dimensions of detected cavities were of few μm, between 1 and 24 μm. It is important to stress that considering the temperature influence on crack growth parameters strongly affects the calculation of the defects’ dimensions; however, a very good agreement between CT data and the back-calculation from CT measurements is obtained. Indeed, the defect size distribution indeed vanishes above 8–10 μm, which corresponds quite well with the value of *c*_0_ of 9 μm previously identified (see inset of Figure 16c).

In addition, further observations could be made by calculating the lifetime based on the evaluated defects. In fact, Equation (10) was used to estimate the lifetime for the two interrupted tests. More specifically, the average *c*_0_ (9 μm) was used as the initial defect size, and the calculated fatigue life for the maximum strains of 5% and 20% were of 3.5 × 10^12^ and 2.9 × 10^8^, respectively. The results are well above the number of cycles at which the tests were stopped (7 × 10^6^ and 2 × 10^6^, respectively). The details concerning the parameters used for the calculation are reported in Table 2.

Similarly, fatigue lifetime predictions were calculated considering the mean values of *c*_0_ and the minimum and maximum values of particle radius found by μ-CT (so 1.7 and 22.2 μm, respectively). Some approximations have been made in the calculations. In particular, the linear relation between the strain energy density *W* and maximum strain (reasonable in the strain range between 50 and 65%) was considered, which resulted in the following equation:

(11) $$W=-757,142+31,014\cdot \varepsilon_{max}.$$

The values of *k* were calculated at the different strains, whereas the material coefficients *m* and *C* were estimated through the aforementioned temperature shifting procedure. A linear relation between temperature and maximum strain was considered:

(12) $$T=21.3+0.62\cdot \varepsilon_{max}.$$

**Figure 17:**
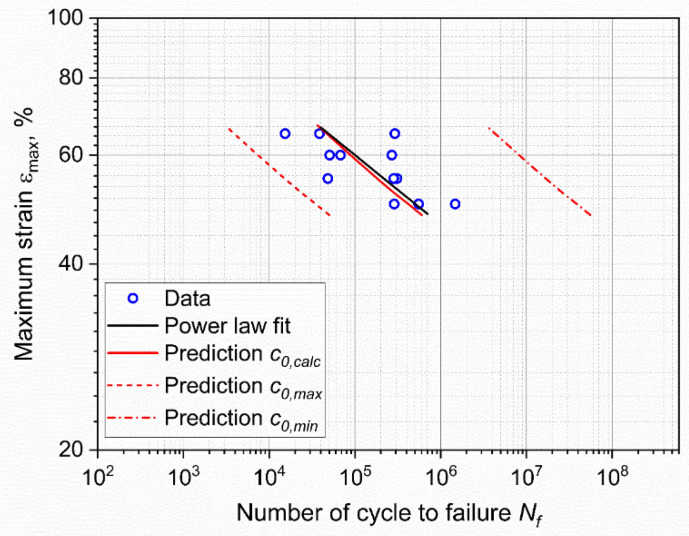
Comparison between fatigue data and the calculated fatigue lifetimes for the calculated defect size (*c*_0,*calc*_), and the maximum and minimum sizes evaluated from μ-CT (*c*_0,*max*_ and *c*_0,*min*_, respectively).

Therefore, the fatigue lifetime predictions were calculated using Equation (10), and these results were then compared with the experimental fatigue results described in the previous section. These results illustrate the impact of initial defect size. As depicted in Figure 17, when considering the minimum and maximum value of particle size, the fatigue predictions show a broad spectrum spanning across three orders of magnitude. Of course, the calculations using the average *c*_0_ give values similar to the experimental data and to the power law fit (Equation (1)): these have been included to illustrate the typical scatter in the data.

### 3.6. Correlation of the Results Based on J-Integral

Finally, a non-linear J-integral hyperelastic model for the CRB has been developed. This energetic approach has the advantage of being applied in order to describe materials that show non-linear mechanical behavior, and it is useful for comparing different geometries. In particular, the J-integral was evaluated as [73]:

(13) $$J=\frac{F^{2}}{\pi {\left(r_{out}-a\right)}^{2}}\cdot f\left(\frac{a}{r_{out}}\right),$$

where *F* is the force, *r_out_* the external radius of the CRB, *a* is the size of notch in the CRB, and *f*(*a*/*r_out_*) is a geometric factor found by the FEM simulation as:

(14) $$f\left(\frac{a}{r_{out}}\right)=11.190{\left(\frac{a}{r_{out}}\right)}^{4}-3.766{\left(\frac{a}{r_{out}}\right)}^{3}-1.072{\left(\frac{a}{r_{out}}\right)}^{2}+3.615\left(\frac{a}{r_{out}}\right)+0.012.$$

**Figure 18:**
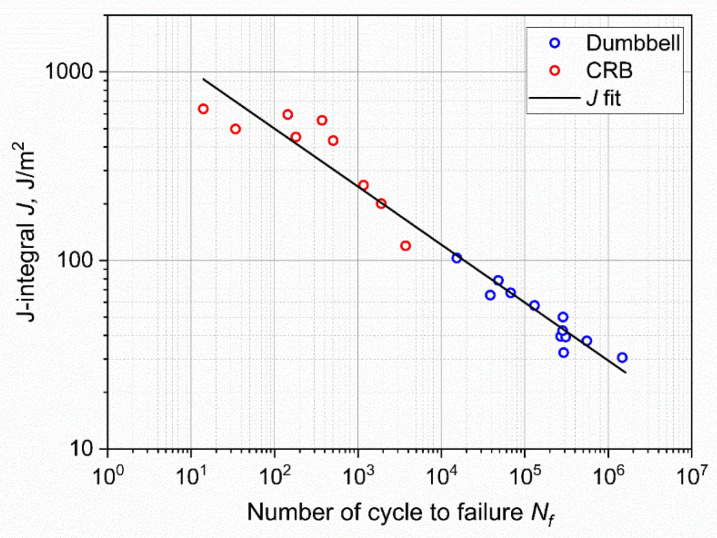
Wöhler curve in terms of the J-integral for axisymmetric dumbbell (blue) and CRB (red) specimens. A unique fitting line was found correlating the two geometries.

Equation (13) was used to compute *J_max_* and *J_min_* using *F_max_* and *F_min_*, respectively. The difference between *J_max_* and *J_min_* was then used to compare the CRB results with those of standard dumbbell samples. The J-integral of the axisymmetric dumbbell was evaluated using Equation (7); in fact, *J* can be approximated as the energy release rate *G* [60]. The values of *J* for dumbbell specimens were evaluated using the calculated *c*_0_ and the strain energies reported in Table 1. The considered forces were those measured at the beginning of the test. An overview of the data used for the evaluation of *J* is reported in Table 3. The J-integral as a function of number of cycles to failure for both geometries is plotted in Figure 18. A unique fitting curve was found for both geometries; this demonstrates that independently of the specimen geometry, it is possible to evaluate the fatigue lifetime based on J-integral. Although this J-integral formulation and its applicability still need to be verified on different geometric parameters and materials, it seems to be a promising candidate for extending fracture mechanic tools for the fatigue assessment of elastomers with reduced testing times.

## 4. Conclusions

The fatigue behavior of a carbon black filled NBR was investigated in the presented work. Fatigue measurements were performed on axisymmetric dumbbell specimens and also on CRB specimens (i.e., axisymmetric dumbbells with a circumferential notch). The number of cycles to failure was measured for both sets, and Wöhler curves were constructed. Although the recorded points were considerably scattered, the results could be described with a power law.

The results of the axisymmetric dumbbell specimens were investigated in more detail through the analysis of temperature variations, hysteresis, secant, dynamic moduli, stored and dissipated energies, and the normalized force peaks. In general, all the observed values largely varied in the early stages of fatigue (approximately up to 2500–3000 cycles), but they stabilized with a lower degree of variation up to the failure of the specimens, suggesting a slow crack growth process. Even though these variations evidenced a continuous and progressive accumulation of relaxation and damage, a clear distinction between initiation and propagation was not observed.

Additional details could be obtained by further investigations considering a fracture mechanics approach. The fracture surfaces featured initiation areas from which evident fatigue striations originated from. Similar aspects of the fracture surfaces were found for axisymmetric dumbbells with a circumferential notch. These results suggested similarities in the damaging mechanism and that the notch can be represented as a larger defect. As expected, the introduction of a notch led to a reduction in fatigue life up to four orders of magnitude.

Moreover, the fatigue life of dumbbells was correlated to the defect size by integrating the crack growth characteristic and taking into account the effect of temperature, obtaining values that were independent on the strain level. The evaluated defect size was then compared with the defect sizes analyzed through X-ray microtomography, confirming similar dimensions and a good accuracy of the calculations.

Finally, through the evaluation of the J-integral, it was possible to link the fatigue life of unnotched and notched axisymmetric dumbbells, evidencing a geometry independency of the J-integral formulation. This formulation for notched axisymmetric dumbbells may represent a new tool for the investigation of accelerated fatigue in elastomers.

## Figures and Tables

**Table 1:** Overview of the values used to calculate the critical defect size with the reversion of Equation (10). The values of the number of cycles to failure are the same as reported in Figure 3.

*ε_max_* (%)	*N_f_*	*W* (kJ/m^3^)	*k* (λ)	*T* (°C)	*m*	*C*
50	1,472,620	715	2.38	47.4	3.7	1.26 × 10^−14^
	551,975	817	2.38	52.7	3.7	1.91 × 10^−14^
	286,860	814	2.38	50.5	3.7	1.62 × 10^−14^
55	48,181	948	2.33	57.4	3.7	2.69 × 10^−14^
	308,772	1000	2.33	56.4	3.7	2.45 × 10^−14^
	283,510	971	2.33	55.0	3.7	2.24 × 10^−14^
60	67,622	1072	2.29	56.2	3.7	2.45 × 10^−14^
	269,073	1103	2.29	57.0	3.7	2.57 × 10^−14^
	130,393	1099	2.29	52.9	3.7	1.91 × 10^−14^
65	291,859	1267	2.26	61.7	3.7	3.63 × 10^−14^
	15,318	1222	2.26	60.3	3.7	3.24 × 10^−14^
	38,475	1288	2.26	63.7	3.7	4.17 × 10^−14^

**Table 2:** Overview of the values used to calculate the number of cycles to failure for interrupted tests using Equation (10).

*ε_max_* (%)	*W* (kJ/m^3^)	*k* (λ)	*T* (°C)	*m*	*C*	*N_f,calc_*
5	16	2.88	26.2	3.7	2.24 × 10^−15^	3.5 × 10^12^
20	187	2.68	31.6	3.7	3.55 × 10^−15^	2.9 × 10^8^

**Table 3:** Overview of the values used to calculate the J-integrals for CRB using Equation (13).

*Ε_max_* (%)	*N_f_*	*a* (mm)	*r_out_* (mm)	*F_max_* (N)	*F_min_* (N)	*J* (J/m^2^)
20	3724	0.47	7.19	526	129	120
	1899	0.69	7.26	556	157	200
	1165	0.86	7.24	540	145	251
30	371	1.00	7.09	702	178	554
	504	0.85	7.29	720	190	433
	180	0.84	7.36	757	206	452
50	14	0.64	7.19	1002	213	638
	144	0.62	7.08	973	229	594
	34	0.71	7.19	841	200	498

## Data Availability

The data presented in this study are available on request from the corresponding author.

## Supplementary Materials

The following supporting information can be downloaded at: https://www.mdpi.com/article/10.3390/ma15113745/s1, Figure S1: Comparison of Wöhler curves considering *N_i_* and *N_f_*.
